# Prognostic value of magnetic resonance imaging in pediatric-onset primary sclerosing cholangitis

**DOI:** 10.1177/17562848261426258

**Published:** 2026-02-27

**Authors:** Enni J. Vanhanen, Tiina E. Lehtimäki, Kaija-Leena Kolho, Andrea Tenca

**Affiliations:** Department of Radiology, Helsinki University Hospital and University of Helsinki, P.O. Box 340, Helsinki 00029, Finland; Faculty of Medicine, University of Helsinki, Helsinki, Finland; Department of Paediatric Gastroenterology, Children’s Hospital, Helsinki University Hospital and Faculty of Medicine, University of Helsinki, Helsinki, Finland; Abdominal Center, Endoscopy Unit, Helsinki University Hospital and Faculty of Medicine, University of Helsinki, Helsinki, Finland

**Keywords:** bile duct, children, cirrhosis, hepatology, liver

## Abstract

**Background:**

The prognostic value of magnetic resonance imaging (MRI) in pediatric-onset primary sclerosing cholangitis (PSC) remains poorly studied. The MRI-based ANALI scores with and without gadolinium (ANALI_Gd_ and ANALI_NoGd_) have predicted disease progression and complications in adults, but these scores have not been studied in children.

**Objectives:**

To assess the prognostic relevance of ANALI scores in pediatric-onset PSC.

**Design:**

This retrospective cohort study was conducted at a single tertiary-care center and included patients with pediatric-onset PSC identified from a national PSC registry.

**Methods:**

We included 34 patients with pediatric-onset PSC (median age at diagnosis 14 years, interquartile range (IQR) 12.0–16.3; median follow-up time 10.4 years, IQR 7.0–13.2) who had undergone at least two MRIs. Two expert radiologists evaluated the presence of intrahepatic duct dilatation, hepatic dysmorphia, collateral veins, and parenchymal enhancement heterogeneity, which were used to calculate ANALI scores for the baseline and follow-up MRIs. The longitudinal development of ANALI scores was assessed, and the association between scores and clinical endpoints (decompensated cirrhosis, liver transplantation) was examined using Cox proportional hazards regression. Interrater agreement was evaluated for the total scores and the individual score items.

**Results:**

Disease progression was variable, and baseline ANALI scores alone did not predict adverse outcomes. When follow-up scores were also assessed, higher scores were associated with outcomes (hazard ratio (HR) per 1-point increase in ANALI_NoGd_ 1.59, 95% confidence interval (CI) 1.15–2.20; *p* = 0.005; HR per 1-point increase in ANALI_Gd_ 3.03, 95% CI 1.06–8.70, *p* = 0.039). Interrater agreement was excellent for ANALI_NoGd_ (0.89, 95% CI 0.83–0.95) and substantial for ANALI_Gd_ (0.73, 95% CI 0.62–0.84).

**Conclusion:**

ANALI scores were associated with adverse outcomes in pediatric-onset PSC when assessed repeatedly during follow-up; baseline measurements alone may have limited prognostic value in the pediatric population.

## Plain language summary

Prognostic value of magnetic resonance imaging in pediatric-onset primary sclerosing cholangitis

This study evaluated the performance of magnetic resonance imaging (MRI) in predicting disease progression and complications in children with a rare liver disease, primary sclerosing cholangitis (PSC). In adult patients with PSC, MRI-based ANALI scores assessed at the time of diagnosis have associated with disease progression and complications, including cirrhosis decompensation and the need of liver transplantation. We examined the performance of ANALI scores in 34 patients with pediatric-onset PSC who had undergone at least two MRIs. Two radiologists examined specific liver features on the scans, and ANALI scores were calculated for the baseline and follow-up MRIs. Findings showed that baseline ANALI scores alone did not predict complications, but when follow-up scores were also assessed, higher scores were associated with outcomes. Our results suggest that ANALI scores are useful for risk stratification in patients with pediatric-onset PSC when assessed repeatedly during follow-up.

## Introduction

Primary sclerosing cholangitis (PSC) is a chronic cholestatic disorder characterized by inflammation and obliterative fibrosis of bile ducts. PSC follows a highly variable but typically progressive course, often culminating in cirrhosis and end-stage liver disease.^1^ PSC also increases the risk of cholangiocarcinoma and gallbladder carcinoma, especially in adults.^1–4^ Currently, there is no curative treatment for PSC, and liver transplantation remains the definitive therapy for end-stage disease.^3^ Due to the variable disease course, reliable prognostic tools are needed to identify high-risk patients and guide follow-up. Magnetic resonance imaging (MRI) has surpassed endoscopic retrograde cholangiopancreaticography (ERCP) as the primary imaging modality for PSC in recent years.^5^ Therefore, it is crucial to identify MRI findings that may predict rapid disease progression and the risk of adverse outcomes, such as decompensated cirrhosis or malignancy. An MRI-based model consisting of separate non-contrast and gadolinium (Gd)-enhanced scores (ANALI_NoGd_ and ANALI_Gd_) that assess liver parenchymal changes, intrahepatic duct (IHD) dilatation, and signs of portal hypertension, has been shown to predict disease progression^6^ and to correlate with cirrhosis and the need for liver transplantation in adults with PSC.^7–12^ However, this model has not been studied in pediatric patients. This study aimed to evaluate the applicability of ANALI scores in predicting disease progression and clinical outcomes in patients with pediatric-onset PSC.

## Methods

### Study design and population

**Figure 1.**
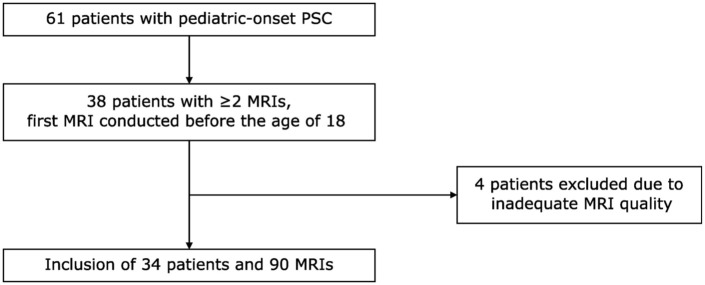
Patient flowchart. MRI, magnetic resonance imaging; PSC, primary sclerosing cholangitis.

This was a retrospective cohort study performed at a tertiary-care center, Helsinki University Hospital, Finland. Patients with pediatric-onset PSC were selected from a national PSC registry held at our center, which includes clinical and therapeutic data as well as biochemical, cholangiographic, and histological findings of patients diagnosed with PSC. The diagnostic criteria followed the guidelines of the European Association for the Study of the Liver,^5^ and patients with secondary sclerosing cholangitis, including IgG4-associated cholangitis, were excluded. Patients diagnosed with PSC before the age of 18 between 1999 and 2017 were identified from the registry. Of these patients, we included those who had at least two MRIs with 3D magnetic resonance cholangiopancreaticography (MRCP) performed between 2001 and the end of June 2023, with a minimum 1-year interval between them, and the first MRI conducted before the age of 18 years. MRIs of insufficient quality or performed after liver transplantation were not included. Clinical outcomes were followed until 5/2025 and included liver transplantation and decompensated liver cirrhosis, defined by the presence of ascites, esophageal varices, or variceal bleeding. A patient flowchart is shown in Figure 1. The research is reported in line with the TRIPOD + AI guideline, where applicable.^13^ A proofreader toolbox of a large language model-based AI tool, Avidnote (Avidemic AB, Gothenburg, Sweden), was used to check the grammar of parts of the manuscript.

### MRI technique

MRIs were performed with 1T (*n* = 1), 1.5T (*n* = 85), or 3T (*n* = 4) MRI scanners. Patients were advised to fast for 3 h before the examination. Before imaging, patients drank pineapple juice to diminish artifacts from bowel contents. The imaging protocol included the following sequences: respiratory-triggered heavily T2-weighted 3D sequence, maximum intensity projection images reconstructed from the 3D sequence, breath-hold thick-slab single-shot heavily T2-weighted sequence in coronal and oblique coronal planes, axial and coronal T2-weighted sequences, axial T2-weighted sequence with fat suppression, and axial in- and out-of-phase T1-weighted gradient echo sequence. When performed, gadolinium-enhanced sequences were acquired with an axial, fat-suppressed T1-weighted sequence before and after 0.2 mL/kg body weight Gd-DOTA (Dotarem^®^; Guerbet, Aulnay-Sous-Bois, France) during the arterial, portal, and equilibrium phase.

### Image analysis

Two abdominal radiologists (E.J.V. and T.E.L., with 3- and 12-year experience in abdominal radiology) independently reviewed the MRIs, blinded to clinical information. Discrepancies between evaluations were resolved through consensus assessment. Images were assessed according to the model described by Ruiz et al.,^6^ including scores with and without Gd. The score without gadolinium (ANALI_NoGd_) was calculated as (1× IHD dilatation + 2× liver dysmorphia + 1× portal hypertension; score total 0–5), and the score with gadolinium (ANALI_Gd_) as (1× liver dysmorphia + 1× parenchymal enhancement heterogeneity; score total 0–2).

Liver dysmorphia, parenchymal enhancement heterogeneity, and portal hypertension were scored as 0 if absent or 1 if present. Liver dysmorphia was defined as significant atrophy of the right or left hepatic lobe, marked lobulations of the liver surface, or an increase in the caudate lobe/right lobe ratio. Parenchymal heterogeneous enhancement was defined as segmental/patchy peripheral or peribiliary enhancement on arterial phase imaging. Criteria for portal hypertension included the presence of collateral veins, with or without accompanying splenomegaly. The maximal IHD dilatation was measured and scored as 0 if it was ⩽4 mm, 1 if it was 4 mm, and 2 if it was ⩾5 mm. In addition, several other liver- and bile duct-related parameters were assessed, in accordance with the study by Ruiz et al.^6^

### Statistical analysis

The association between ANALI scores and clinical endpoints was assessed using Cox proportional hazards regression analysis. The first analysis followed the methodology of comparable studies conducted in adult cohorts^7–12^ and included only the baseline ANALI score. Data were censored at the date of last visit (until 5/2025) or at the time of adverse outcome. Outcome-free survival was visualized using Kaplan–Meier analysis (with patients grouped by ANALI_NoGd_ baseline score 0–1 and 2–5, and ANALI_Gd_ 0 and 1–2), and differences between survival curves were tested with the log-rank test. In addition, we conducted a second analysis utilizing longitudinal follow-up data on the ANALI score collected in this study. For this, we fitted a Cox proportional hazards model in counting-process form to incorporate a time-dependent covariate (the ANALI score). The analysis was done using the survival package^14,15^ in R, version 4.4.1.^16^ *p* < 0.05 was considered significant.

Interrater agreement was assessed individually for ANALI_NoGd_ and ANALI_Gd_ scores, as well as for each individual item (IHD dilatation, hepatic dysmorphia, collaterals, parenchymal enhancement heterogeneity) with weighted kappa using quadratic weights or with Cohen’s kappa, as appropriate. Continuous variables are expressed as medians with interquartile ranges (IQR) unless otherwise stated. Considering the retrospective nature of this pediatric cohort, we did not apply imputation for missing data.

### Ethical considerations

The study was registered to the Helsinki University Hospital Research Portal (number 64/13/03/03/2012). All research was conducted in accordance with the Declaration of Helsinki. According to Finnish legislation, informed patient consent or approval by the institutional ethics committee were not required because the patients were not contacted in this register-based study. There was no patient or public involvement during the study.

## Results

### Patient characteristics

The analysis included 34 patients with pediatric-onset PSC with a total of 90 MRIs and 366 patient-years. In 25 patients (74%), the first MRI was performed for diagnostic purposes. In the remaining patients, the median time interval between diagnosis and the first MRI was 3.0 years (IQR 1.3–3.8). Patient characteristics are shown in Table 1.

**Table 1.** Patient characteristics.

Variables	*N* = 34
Female	13 (38%)
Age at PSC diagnosis	14.0 (12.0–16.3)
Age at first MRI	15.0 (12.9–16.1)
Age at last follow-up	23.9 (21.0–27.6)
Interval between first and last MRI (years)	7.2 (4.2–10.7)
Interval between first MRI and adverse outcome/last follow-up	10.4 (7.0–13.2)
PSC with features of autoimmune hepatitis	9 (26%)
IBD	28 (82%)
• Ulcerative colitis	20 (71%)
○ Pancolitis (E4)	18
○ Left-sided (E2)	1
○ Proctitis (E1)	1
• Crohn’s disease	8 (29%)
○ Pancolitis (L2)	3
○ Ileocolonic (L3)	3
○ Gastroesophageal + colonic (L4a + L2)	1
○ Not determined	1
• Intestinal dysplasia	1
• IBD-related surgery	7
Other autoimmune disease	6 (18%)
Medication at the time of the first MRI	
• Ursodeoxycholic acid	23 (68%)
• Vancomycin	1 (2.9%)
• Immunosuppressant	7 (21%)
• Biological medication	2 (5.8%)
• Glucocorticoid	11 (32%)
Laboratory values at the first MRI	
• AST U/L (*n* = 28)	41 (25–76)
• ALT U/L (*n* = 31)	39 (21–90)
• GT U/L (*n* = 27)	107 (29–158)
• ALP U/L (*n* = 26)	218 (151–293)
• Bil µmol/L (*n* = 28)	6 (5–9)
• Alb g/L (*n* = 28)	37 (36–39)
• Tromb E9/L (*n* = 31)	321 (245–392)
• APRI (*n* = 28)	0.32 (0.17–0.64)
Biliary intervention during follow-up	
• Right hepatic duct dilatation	9 (26%)
• Left hepatic duct dilatation	12 (35%)
• Common hepatic duct dilatation	13 (38%)
• Common bile duct dilatation	10 (29%)
• Stent	8 (24%)
Outcome	
• Decompensated cirrhosis	3 (12%)^a^
• Liver transplantation	5 (18%)

Continuous variables are expressed as medians with IQRs. Events during the whole follow-up period are included. Other autoimmune diseases included rheumatoid arthritis, nephritis, asthma, psoriasis, thyroiditis, and type 1 diabetes. Ulcerative colitis and Crohn’s disease were classified according to the Paris classification of IBD.^17^

a One patient presented with esophageal varices at baseline MRI; however, the patient was retained in the study cohort and monitored with respect to progression to liver transplantation (esophageal varices at baseline were not considered as an outcome).

Alb, albumin; ALP, alkaline phosphatase; ALT, alanine aminotransferase; APRI, aspartate aminotransferase to platelet ratio index; AST, aspartate aminotransferase; Bil, bilirubin; GT, gamma-glutamyl transferase; IBD, inflammatory bowel disease; MRI, magnetic resonance imaging; PSC, primary sclerosing cholangitis; Tromb, thrombocytes.

### Disease characteristics on the first and last MRI

On the first MRI, all patients had strictures in IHDs, and 23 patients (68%) had at least one severe stricture (⩾75% luminal narrowing). IHD changes were diffuse in 28 patients (82%), and 11 patients (32%) had upstream dilatation, which was marked (⩾5 mm) in 5/11 (45%). Fifteen patients (44%) had strictures in the common bile duct, severe in 8/15 (53%), and confluent (⩾10 mm) in 10/15 (67%). Six patients (18%) showed changes in liver morphology, and four patients (12%) had collaterals as a sign of portal hypertension.

On the last MRI, all patients had strictures in IHDs, and 31 patients (91%) had at least one severe stricture. IHD changes were diffuse in all patients, and 13 patients (38%) had upstream dilatation, which was marked in 8/13 (62%). Seventeen patients (50%) had strictures in the common bile duct, severe in 8/17 (47%), and confluent in 12/18 (67%). Fifteen patients (44%) had liver dysmorphia, and 10 patients (29%) had collaterals.

### ANALI_NoGd_ score in the evaluation of disease stage and progression

**Figure 2.**
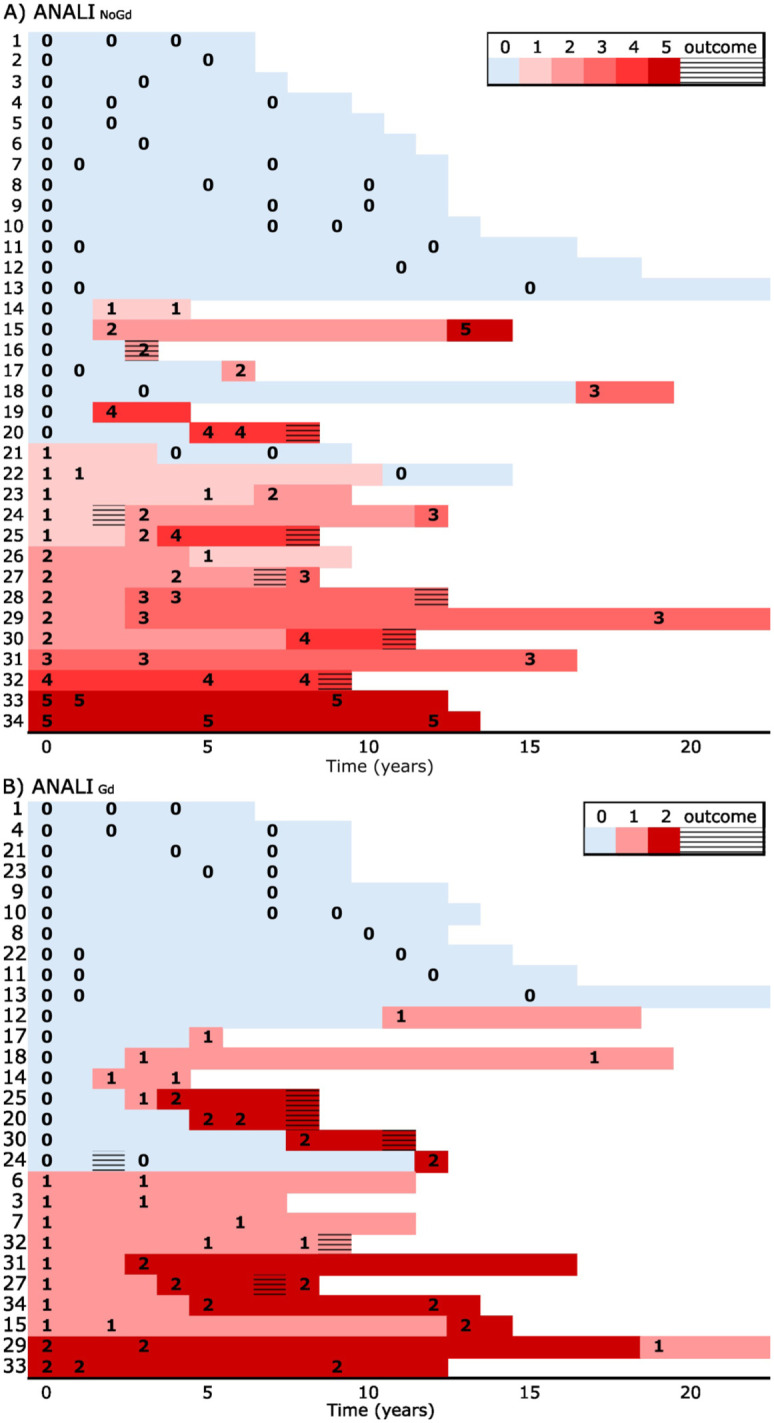
(a) ANALI_NoGd_ and (b) ANALI_Gd_ scores at baseline and during follow-up. Outcome (striped box) was defined as cirrhosis decompensation or liver transplantation. The color corresponding to the last observed score is extended until the occurrence of an endpoint or the end of follow-up. Median follow-up time was 10.4 years (IQR: 7.0–13.2). IQR, interquartile range.

ANALI_NoGd_ scores were calculated for all patients (total: 90 MRIs). Figure 2 illustrates the scores at baseline and during follow-up, and Table 2 presents the distribution of the individual parameters across the different score categories.

**Table 2.** Distribution of parameters of ANALI_NoGd_ score across the different score categories.

Score	Findings
1	IHD dilatation (10 MRIs)
2	IHD dilatation (7 MRIs)
	Dysmorphy (4 MRIs)
	IHD dilatation and collaterals (1 MRI)
3	Dysmorphy and collaterals (7 MRIs)
	Dysmorphy and IHD dilatation (1 MRI)
4	Dysmorphy and IHD dilatation (4 MRIs)
	Dysmorphy and collaterals and IHD dilatation (4 MRIs)
5	Dysmorphy and collaterals and marked IHD dilatation (7 MRIs)

IHD, intrahepatic duct; MRI, magnetic resonance imaging.

The score was stable in 17 patients (50%). Among these patients, the score remained at 0 in 13/17 patients (median interval between the first and last MRI: 7.2 years). Two patients exhibited the maximum score already at baseline, while one maintained a score of 3 and another a score of 4 throughout the follow-up. In 14 patients (41%), the score increased during the follow-up. The rate of disease progression varied among patients: five patients maintained a score of 0 for at least 10 years, whereas three patients exhibited rapid progression, with ANALI_NoGd_ score increasing by ⩾1 score point per follow-up year.

**Figure 3.**
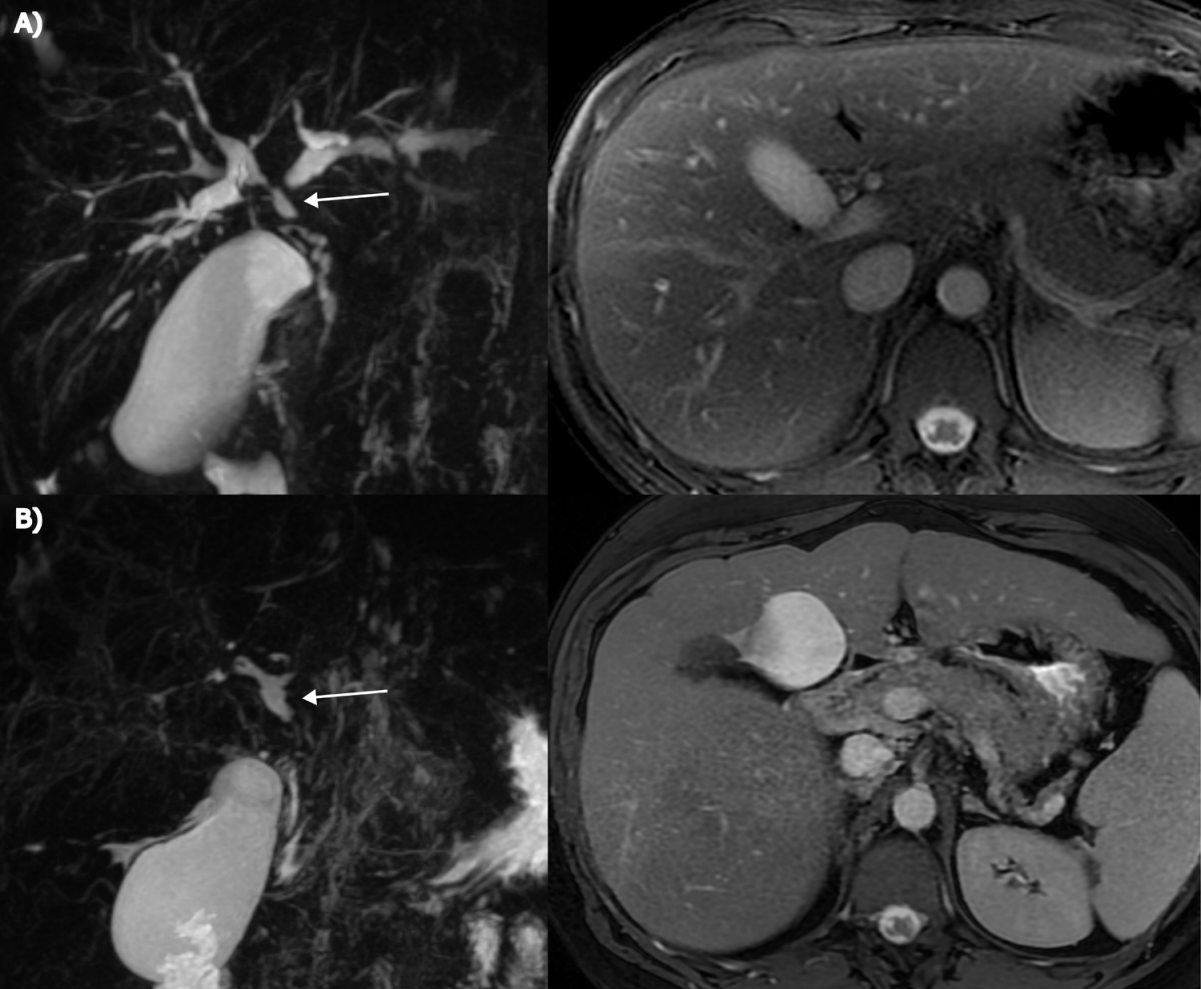
An example of a counter-intuitive behavior of ANALI subscores. (a) Patient presenting with marked IHD dilatation and normal liver morphology on initial MRI (ANALI_NoGd_: 2 + 0 + 0 = 2). (b) After an 8-year follow-up period, the IHD dilatation had decreased, while extensive biliary stricturing with a pruned-tree appearance had developed, accompanied by progressive hepatic dysmorphia and the formation of collateral vessels (not shown). Despite noticeable disease progression, the ANALI_NoGd_ score increased only to 3 due to diminished IHD dilatation (ANALI_NoGd_: 0 + 2 + 1 = 3). For anatomical orientation, the common hepatic duct is indicated by an arrow in the figures. IHD, intrahepatic bile duct.

In three patients (9%), the score decreased during the follow-up due to diminished IHD dilatation (from 1 to 0 in two patients, from 2 to 1 in one patient). The MRIs of these patients were re-evaluated, revealing progressive stricturing in the follow-up scans, accompanied by milder IHD dilatation. However, no changes in liver morphology or collaterals were observed. Decreasing IHD dilatation was also seen in three patients with stable or increasing total scores (Figure 3). Additionally, three patients presented with advanced disease findings (abnormal liver morphology and collaterals), but never developed IHD dilatation. Abnormal liver morphology and collaterals, once developed, persisted in all patients.

### ANALI_Gd_ score in the evaluation of disease stage and progression

Two or more gadolinium-enhanced MRIs were available for 28 patients (82%), with a mean of 2.7 MRIs per patient and a total of 75 MRIs. Figure 2 shows the scores at baseline and during follow-up. Of the 28 patients with serial gadolinium-enhanced MRIs, the score was stable in 15 patients (54%), increased in 12 patients (43%), and decreased in 1 patient (4%). Among patients with stable disease, the score remained at 0 in 10 patients (median interval between the first and last MRI: 8.4 years). One patient had a score of 2 already at baseline, and four patients maintained a score of 1 throughout the study.

Arterial phase heterogeneous enhancement was observed in 12/28 patients, and liver dysmorphia in 14/28 patients. In four patients, arterial phase heterogeneous enhancement was seen before the development of liver dysmorphia, and in two patients, it appeared after liver dysmorphia. In 2/12 patients, arterial phase heterogeneous enhancement disappeared during follow-up. This explains the decrease in the score points observed in one patient during follow-up. Liver dysmorphia, once developed, persisted in all 14/28 patients.

### Concordance between the ANALI scores

ANALI_NoGd_ score and ANALI_Gd_ scores showed consistent behavior in 21 out of 28 patients (75%), while in 7 patients (25%), the scores evolved differently during follow-up (Figure 2). In three patients, ANALI_Gd_ score increased during follow-up due to the appearance of arterial phase heterogeneous enhancement, whereas ANALI_NoGd_ score remained stable. In two patients, ANALI_Gd_ score remained stable, but ANALI_NoGd_ score decreased due to diminishing IHD dilatation. In one patient, the ANALI_NoGd_ score increased due to increasing dilatation, while the ANALI_Gd_ score was stable. Finally, in one patient, ANALI_NoGd_ score increased due to the development of collaterals in addition to liver dysmorphia, whereas ANALI_Gd_ score decreased as the arterial phase heterogeneous enhancement disappeared.

### Prognostic performance of ANALI scores

The Cox model with time-dependent ANALI_NoGd_ score was based on 87 risk intervals and 8 observed outcomes. ANALI_NoGd_ score was associated with the hazard of the outcome (hazard ratio (HR) per 1-point increase, 1.59; 95% CI, 1.15–2.20; *p* = 0.005), with a concordance of 0.75 (SE = 0.07). For the ANALI_Gd_ score, the model was based on 72 risk intervals and 6 outcomes, yielding an HR of 3.03 per 1-point increase (95% CI, 1.06–8.70; *p* = 0.039); concordance was 0.70 (SE = 0.12).

**Figure 4.**
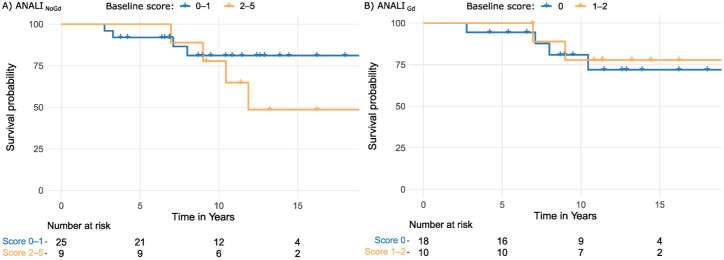
Kaplan–Meier curves for adverse outcome-free survival probability for baseline (a) ANALI_NoGd_ and (b) ANALI_Gd_ scores. The blue line represents patients with low scores (0–1 for ANALI_NoGd_ and 0 for ANALI_Gd_) and the orange line patients with high scores (2–5 for ANALI_NoGd_ and 1–2 for ANALI_Gd_). Adverse outcome was either occurrence of decompensated cirrhosis or liver transplantation. *X*-axis is truncated at the earliest time with ⩽10% of the cohort remaining at risk.

For baseline ANALI_NoGd_ and ANALI_Gd_ scores, univariate Cox-regression analysis revealed an HR of 1.15 (95% CI, 0.78–1.70), with a concordance of 0.59 (SE = 0.09) and an HR of 0.66 (95% CI, 0.16–2.70) with a concordance of 0.55 (SE = 0.09), respectively. Neither of these were statistically significant (*p* = 0.49 and *p* = 0.56). Kaplan–Meier visualization of outcome-free survival by grouped ANALI_NoGd_ scores revealed a trend that patients with a lower initial score had longer outcome-free survival, but this was not statistically significant (log-rank test *p* = 0.28). The baseline ANALI_Gd_ score was not associated with outcome-free survival (*p* = 0.75; Figure 4).

### Interrater agreement

Interrater agreement for ANALI_NoGd_ was 0.89 (0.83–0.95) and for ANALI_Gd_ 0.73 (0.62–0.84). Interrater agreement for IHD dilatation was 0.84 (0.74–0.95), for liver dysmorphia 0.89 (0.78–1.0), for collaterals 0.49 (0.25–0.74), and for parenchymal enhancement heterogeneity 0.39 (0.21–0.56).

## Discussion

This retrospective study assessed the applicability of a prognostic MRI-based model (ANALI scores),^6^ originally developed for adults, in a pediatric PSC cohort with longitudinal data. Disease progression was variable, and baseline ANALI scores alone did not predict outcome within the median follow-up of 10 years. However, when longitudinal ANALI score follow-up data were also considered, a higher ANALI score was significantly associated with a higher risk of progression to decompensated cirrhosis and liver transplantation. IHD dilatation, one of the parameters of the ANALI_NoGd_ score, decreased in some patients during follow-up despite evidence of disease progression. Interrater agreement was excellent for ANALI_NoGd_ and substantial for ANALI_Gd_.

ANALI scores were developed by Ruiz et al., who examined the significance of various biliary and liver parenchymal findings for radiological progression. Altered liver morphology, signs of portal hypertension, heterogeneous parenchymal enhancement, and intrahepatic bile duct dilatation showed the strongest association with disease progression, and ANALI scores are based on these findings. In that study, disease progression occurred rapidly, and nearly 90% of patients with progressive disease had higher initial scores, whereas almost 85% of patients with low scores exhibited stable disease.^6^ Later on, ANALI scores have demonstrated good prognostic performance in several studies involving adult patients, with a low baseline native score (0–2) being associated with a more favorable prognosis than higher scores (3–5).^7–10,12^

In our study, patients with an initially low ANALI score demonstrated heterogeneous disease trajectories, with progression rates ranging from slow to more rapid over time. Even in patients with a baseline native score of 0 or 1, disease progression occurred in 24% within 3 years of follow-up. In addition, several patients with low baseline scores experienced an adverse outcome during follow-up. While baseline ANALI scores alone did not predict outcomes, analyses incorporating ANALI scores as time-varying covariates demonstrated a significant association between higher scores during follow-up and the risk of progression to decompensated cirrhosis and liver transplantation. These findings suggest that changes in ANALI scores over time may capture clinically relevant disease dynamics that are not reflected by a single baseline measurement, and repeated assessment of ANALI scores during follow-up may be more informative for risk stratification than baseline measurements alone. Pediatric PSC has been reported to be milder^18^ and associated with a more favorable prognosis than adult-onset disease,^1,19,20^ and it is possible that the less severe disease phenotype explains why baseline scores alone may not reliably predict long-term disease course.

In our patient cohort, a typical radiological finding at disease onset was IHD dilatation without changes in liver morphology or collaterals. In progressive disease, morphological liver damage became apparent, accompanied by the development of collateral vessels. However, IHD dilatation decreased in 18% of patients during follow-up. The observed decrease in IHD dilatation could be explained either by ERCP with mechanical dilatation of extrahepatic strictures, performed between MRI examinations in 67% of these patients, or by disease progression. Notably, three patients who initially had or later developed liver dysmorphia and collateral vessels never exhibited IHD dilatation—consequently, patients with advanced disease could still have a maximum score of only 3, as shown in Figure 3. Thus, IHD dilatation appears to be a somewhat problematic scoring parameter, as its level may vary during disease progression, and it is typically absent in the end-stage “pruned-tree” pattern with extensive obliterating fibrosis of the bile ducts.^21^ In previous studies, the prognostic significance of bile duct dilatation has been variable. One study reported only a weak association with clinical endpoints,^11^ whereas two studies found a good correlation between bile duct dilatation and clinical outcomes.^22,23^

In the ANALI score with gadolinium, the arterial phase heterogeneous enhancement disappeared in the follow-up in 2/12 patients. In some instances, heterogeneous enhancement may reflect transient secondary cholangitis rather than changes attributable to the primary disease. Moreover, the evaluation of this parameter is not clearly defined, and this may challenge its interpretation—in two previous studies, inter-rater agreement for parenchymal enhancement heterogeneity ranged from poor to moderate.^11,12^ In our study, agreement was similarly limited, with only fair concordance. In the study by Ruiz et al., none of the patients showed improvement in disease status when the total sum of raw parameters was compared between the beginning and end of follow-up. In some patients with progressive disease, however, the raw scores fluctuated during the follow-up. The study did not provide a detailed analysis of the behavior of individual parameters, nor did it present the longitudinal trends of the composite ANALI scores with and without gadolinium.^6^

To our knowledge, two earlier studies have investigated the prognostic value of MRI findings in pediatric-onset PSC.^24,25^ While ANALI scores emphasize parenchymal abnormalities and signs of portal hypertension, these studies focused on biliary tract changes. In a study by Patil et al.^24^ including 45 children, MRCP findings at the time of diagnosis were assessed using the Majoie score,^26^ originally developed for ERCP. A composite score representing the sum of the most severely affected intrahepatic and extrahepatic regions had the best correlation with decompensated cirrhosis, cholangiocarcinoma, and liver transplantation. In that study, liver parenchymal changes had less prognostic relevance than the findings in the bile ducts. Nevertheless, the follow-up period in that study was limited, with a median duration of 3.4 years, and to our knowledge, external validation of their scoring system is still lacking.^24^ MRCP is also a part of the SCOPE-index, a composite score of biochemical parameters and cholangiography findings developed in a multicenter study involving over 1000 pediatric patients with PSC.^25^ In this study, MRCP findings were roughly categorized as positive or negative, and their prognostic significance was less pronounced than that of the clinical parameters included in the scoring system.

Beyond ANALI scores, the DisStrict score—based on the severity and extent of bile duct changes—was introduced in adult patients with PSC and demonstrated a good predictive value for clinical outcomes in a retrospective study of 220 individuals.^27^ Furthermore, several studies have shown that novel MRCP+ software that focuses on changes in MRCP yields reproducible results and correlates with clinical outcomes in adult patients.^22,23,28,29^ In contrast, in the study by Ruiz et al.,^6^ intra- or extrahepatic strictures were not associated with prognosis in multivariate analysis. In another study, biliary duct changes did not correlate with the clinical risk assessment system, Mayo Risk Score.^30^ We did not assess the association between biliary strictures and clinical endpoints. Notably, MRCP sequences are sensitive to various artifacts (e.g., motion and gas) that can impair interpretation. Also, even with optimal technical quality, the evaluation and measurement of small structures remain imprecise.^31^ However, although the sequences used to evaluate parenchymal findings are less sensitive to artifacts, several studies^9–12^ have reported only moderate interrater agreement for ANALI scores, possibly due to the subjectivity of the score parameters. In our study, however, the interrater agreement for ANALI_NoGd_ was almost perfect, and for ANALI_Gd_ substantial.

Our study has several strengths, including a long median follow-up time of 10 years, allowing follow-up until adulthood. Most baseline MRIs were performed at the time of diagnosis, providing valuable insight also into the early phase of the disease. Two expert radiologists reviewed the MRIs independently, and interrater agreement was calculated for the total ANALI scores and the individual score parameters. Also, to our knowledge, this is the first study to assess ANALI scores longitudinally. The limitations of our study include its retrospective nature and the limited number of patients and outcomes, although comparable to previous studies on pediatric PSC. Due to the retrospective nature of the study, MRIs were acquired on several different MRI scanners with varying image protocols. Importantly, all MRIs met the quality requirements, including a single MRI performed with a 1 Tesla MRI scanner.

## Conclusion

Unlike findings in adult populations, baseline ANALI scores alone did not predict disease course or adverse outcomes in this pediatric-onset PSC cohort. In contrast, incorporating ANALI scores obtained during follow-up revealed that higher scores were associated with the development of clinical outcomes. These findings suggest that repeated assessment of ANALI scores provides more informative insight into disease dynamics and the risk of adverse outcomes than a single baseline measurement. Moreover, our IHD dilatation, one of the parameters of the ANALI_NoGd_ score, appears to be a problematic scoring parameter, as its level may vary during disease progression, and it is typically absent in the end-stage liver disease. Based on our results, ANALI scores show good interrater reliability in the pediatric population and are most informative for risk stratification when evaluated repeatedly over time.

## Declarations

**Ethics approval and consent to participate:** The study was registered with the Helsinki University Hospital Research Portal (number 64/13/03/03/2012). All research was conducted in accordance with the Declaration of Helsinki. There was no patient or public involvement during the study. Consent to participate: According to Finnish legislation, informed patient consent or approval by the institutional ethics committee was not required because the patients were not contacted in this register-based study.

**Consent for publication:** Not applicable.

**Availability of data and materials:** Ethical restrictions prevent the authors from making raw data publicly available. However, the relevant summary tables of the data and the analytical code are available upon reasonable request, and with the approval of the hospital’s research ethics committee.
